# Role of Electrophysiology in the Early Diagnosis and Follow-Up of Diabetic Retinopathy

**DOI:** 10.1155/2015/319692

**Published:** 2015-05-05

**Authors:** Nicola Pescosolido, Andrea Barbato, Alessio Stefanucci, Giuseppe Buomprisco

**Affiliations:** ^1^Department of Cardiovascular, Respiratory, Nephrologic, Anesthesiologic and Geriatric Sciences, Faculty of Medicine and Dentistry, “Sapienza” University of Rome, Viale del Policlinico 155, 00161 Rome, Italy; ^2^Center of Ocular Electrophysiology, Department of Sense Organs, Faculty of Medicine and Dentistry, “Sapienza” University of Rome, Viale del Policlinico 155, 00161 Rome, Italy; ^3^Faculty of Medicine and Dentistry, “Sapienza” University of Rome, Viale del Policlinico 155, 00161 Rome, Italy; ^4^Department of Sense Organs, Faculty of Medicine and Dentistry, “Sapienza” University of Rome, Viale del Policlinico 155, 00161 Rome, Italy

## Abstract

Retinopathy is a severe and common complication of diabetes, representing a leading cause of blindness among working-age people in developed countries. It is estimated that the number of people with diabetic retinopathy (DR) will increase from 126.6 million in 2011 to 191 million by 2030. The pathology seems to be characterized not only by the involvement of retinal microvessels but also by a real neuropathy of central nervous system, similar to what happens to the peripheral nerves, particularly affected by diabetes. The neurophysiological techniques help to assess retinal and nervous (optic tract) function. Electroretinography (ERG) and visual evoked potentials (VEP) allow a more detailed study of the visual function and of the possible effects that diabetes can have on the visual function. These techniques have an important role both in the clinic and in research: the central nervous system, in fact, has received much less attention than the peripheral one in the study of the complications of diabetes. These techniques are safe, repeatable, quick, and objective. In addition, both the ERG (especially the oscillatory potentials and the flicker-ERG) and VEP have proved to be successful tools for the early diagnosis of the disease and, potentially, for the ophthalmologic follow-up of diabetic patients.

## 1. Introduction

Diabetes mellitus (DM) is a metabolic consequence of a decrease in insulin production and/or activity characterized by hyperglycemia and vascular and nerve impairment. The macroangiopathy and, above all, the microangiopathy are the most important pathogenic consequences of the excess of glucose in the blood. We can distinguish two main types of diabetes: type 1 diabetes (T1D) in which the main cause is a deficiency of insulin production due to self-destruction of the pancreatic beta-cells and type 2 (T2D) in which the initial insulin resistance leads, with time, to an insulin deficiency.

Diabetic retinopathy (DR) is a serious and frequent complication of diabetes resulting from damage to the retinal microvasculature. The retinal cells primarily involved in DR are both endothelial and neuronal cells. With time, especially if the glycemic control is not adequate, diabetes causes a weakening of the walls of smaller vessels that results in the formation of microaneurysms and then edema, bleeding, and microinfarcts (ischemia). The next stage of retinopathy is called “proliferative,” because neovascularization occurs. The new vessels grow in a chaotic way by destroying nervous tissue, causing increasingly serious bleeding and promoting retinal detachment.

The prevalence of DR is directly proportional to the prevalence of DM [1]. To date, approximately 366 million people worldwide have diabetes and this number is expected to increase. The incidence of the disease is increasing exponentially in developing countries [2, 3].

DR is generally considered a disease of the retinal vessels but has been rarely approached as a real neurosensory disorder [4] in which the visual impairment results not only from a microvascular alteration but also from a nervous impairment (“diabetic encephalopathy”). Ocular symptoms (such as a slow and gradual decrease in visual acuity, metamorphopsia, and a sudden loss of vision in one eye) occur when the DR has reached a very advanced and irreversible stage: the diagnosis is often too delayed. Currently it requires an eye examination with a careful ocular funduscopy. In certain cases there is indication for specific techniques such as the optical coherence tomography or OCT (in particular in the presence of a macular edema) and the intravenous fluorescein angiography (IVFA), which, however, is an invasive examination (it needs an intravenous injection of a contrast medium).

In recent years, psychophysical and electrofunctional exams are having an increasing use because several studies have shown the sensitivity of these methods in identifying signs of the disease already in the preclinical phase.

Over the past two decades, the advent of new neurophysiological techniques to assess retinal and brain (optic tract) function, such as electroretinography (ERG) and the measurement of visual evoked potentials (VEP), allowed a more detailed study of the visual function and of the effects that DM can have on it.

## 2. The Standard Electroretinogram (ERG)

The standard flash ERG is an electrofunctional test able to evaluate the bioelectrical massive retinal response to an unstructured light stimulus (flash). It allows us to test the operation of the entire surface of neuroretina, limited to the photoreceptor and outer plexiform layers. The potentials recorded reflect many events that relate to different types of cells: photoreceptors, bipolar cells, amacrine cells, and Müller cells.

According to the International Society for Clinical Electrophysiology of Vision (ISCEV) [5], the standard ERG examination (Table 1) consists of a minimum of 5 different surveys: scotopic ERG (dark adapted eye and weak flash), massive combined ERG (dark eye and strong flash), oscillatory potentials, photopic ERG (ERG cone with strong flash and light adapted eye) and flicker-ERG (with a quickly repeated stimulus). Each component of the ERG is characterized by the following parameters: the latency (the time that elapses between the start of the stimulus and the beginning of the response), the implicit time (the time, expressed in milliseconds, between the start of the stimulus and the peak of the response), and then the amplitude (e.g., the voltage wave).

Tzekov and Arden already in 90s emphasized the importance of light adapted flash ERG and oscillatory potentials in understanding the pathophysiology of DR and light adapted flash ERG and oscillatory potentials usefulness in predicting progression from nonproliferative to the more sight-threatening stages (preproliferative or proliferative) [6].

In a research of Yamamoto et al. flash ERG has been used to study the responses of cones in 31 diabetics (15 of them had no signs of retinopathy) [7]. Results showed, in diabetics with or without retinopathy, an early involvement of type S cones sensitive to blue light (the amplitude of the b-wave was reduced) which appear to be more susceptible to hypoxic damage [8].

However, oscillatory potentials (OPs) are considered the most relevant electroretinographic test for DR diagnosis [9]. They are 4/5 waves of small amplitude and high frequency that overlap the ascending phase of the b-wave [10, 11]. These waves seem to reflect the activity of the negative feedback exerted by the amacrine cells towards bipolar and ganglion cells. The oscillatory potentials are excellent markers of trophic disorders of the retina and, therefore, frequently they are absent in diabetic patients even in a preclinical stage of retinopathy [4, 12]. OP-2 and OP-3, in particular, tend to disappear early when the foveal and parafoveal area are affected while OP-4 disappears in more extensive injuries [13].

Luu et al. [14], in an attempt to correlate the changes in the ERG with the caliber of the retinal vessels of patients without clinical signs of DR, have shown a reduction in the amplitude of the oscillatory potentials and slower implicit time; the scotopic ERG has also allowed them to detect a predominant involvement of the rods.

An increase in the activity of Müller cells has been demonstrated in mice with streptozotocin-induced diabetes (the streptozotocin is a substance toxic to pancreatic beta-cells; a single injection of 60–70 mg/kg is sufficient to cause an insulin-dependent diabetes in 48 hours). This phenomenon resulted in an alteration of OPs, a reduction of amplitude, and an increase in latency [15]. Using the same type of laboratory animals, in 2011 Wright et al. [16] have postulated the possible role of glutathione (GSH) in the genesis of electroretinographic alterations: indeed there were noted correlations between GSH and all ERG parameters (with the exception of b-wave implicit times), not significantly altered by the presence of hyperglycemia.

## 3. The Flicker-ERG

Neurovascular coupling is a physiological process adjusting the nervous microcirculation blood flow in response to neuronal activity. The flicker-ERG stimulation (30 Hz frequency) was used in healthy subjects to study this process: indeed, it induces a greater activity of nerve cells and, therefore, a microvascular response due to release of NO (nitric oxide) and other vasodilatory substances by excited neurons and by endothelial cells [17–20].

Several studies, using instruments able to evaluate the response of retinal vasculature as the Dynamic Vessel Analyzer (DVA; IMEDOS, Jena, Germany), have shown that, in diabetic subjects without signs of retinopathy, there is a reduction of the retinal vessels vasodilator capacity in response to flicker stimulation [21, 22]. Probably this is the basis of the reduced oxygen supply to the retina in diabetic subjects [23] and this impairment seems to be directly proportional to the degree of retinopathy, if it is present [24].

Therefore, at the genesis of altered responses to flicker stimulation in diabetic subjects, several mechanisms seem to contribute [25]: on the one hand there is the damage to neurons and photoreceptors; on the other hand there is the microvascular damage itself, which (causing hypoxic injury) establishes a sort of vicious cycle against the retinal cells.

Recently, the Miniganzfeld stimulator RETIMAX by CSO (Scandicci, Florence, Italy) is under implementation with specific analytical software for DR (diabetic retinopathy test, DRT). The DRT is based on 30 Hz flicker stimulation and allows evaluating both the amplitude and latency showing, for each parameter, the standard deviation (SD) compared to normative values present in a database (based on the age of the patient). Further studies about this test are currently underway.

## 4. The Multifocal-ERG (mfERG)

The mfERG is considered the best electrofunctional method to diagnose and monitor macular disorders [26]. It provides a measure of retinal and macular integrity especially when the changes are minimal and dysfunction is localized in a small area. The mfERG reflects the function of a wide part of the posterior pole (40–50 degrees), and the result obtained groups together a set of weak amplitude responses (10^−9^ volts) mainly elicited by the first two retinal layers (photoreceptor layer and outer plexiform-bipolar layer) [27].

Searching some predictive risk factors for the development of DR, numerous research groups have used the mfERG. The reason for this interest is the discovery that in the retina occur neuronal alterations (and thus functional ones) much earlier than vascular impairment, which is already an indication of anatomical damage: mfERG allow correlating very accurately a functional deficit with the part of retina affected [28]. The parameters considered in the various studies have been the implicit time (IT) and the amplitude (AMP) of P1 main wave.

Among the most relevant studies in this sense, Harrison et al. [29] showed the sensitivity of mfERG in the early detection of retinal areas affected by DR, correlating functional alterations (increase of IT and reduction of amplitude) with the anatomical damage. They monitored 46 eyes of 23 patients using a grid dividing retina into 35 zones: the most altered areas at mfERG examination were, during the follow-up, the first to develop a macular edema.

Similar approach, but with a longer lasting follow-up, had the study of Ng et al.: [30] results found were comparable even with a lower sample size of subjects examined (18 patients).

Recently Laron et al. [31], evaluating mfERG in younger people (adolescents with T1D), observed an increased susceptibility (and particularly an increased IT) of the nasal retina compared to other areas, as also Holm and Adrian have demonstrated in adults [32]: these findings indicate that the nasal retinal area is the most vulnerable to diabetic damage, and mfERG can be very useful for early evaluation.

Similar target study in 2010 of Lakhani et al. [33] examined mfERG in 48 adolescents with T1D without DR and 45 controls. Considered parameters were glycated hemoglobin (HbA1c) levels, time since diagnosis of diabetes, age at diagnosis, age at testing, and sex. The researchers recorded standard (103 hexagons) and slow-flash (61 hexagons) mfERG and found that a poor long-term glycemic control is associated with an increase of localized neuroretinal dysfunction areas.

Therefore, latest researches have demonstrated that mfERG reveals local retinal dysfunction in diabetic eyes before the onset of retinopathy, in direct proportion to the degree of clinical abnormality. In particular, the analysis of P1 IT variations improves the test sensitivity since is the first parameter to be altered [34]. Hard exudates, especially, seem to prolong P1 implicit time compared to healthy eyes and independently of macular thickness [35].

Other authors did comparisons with particular programs, as the M1M2 paradigm [36] or the photopic negative response [37], and, even in these cases, the ability of the mfERG to identify the damaged areas of the retina in the preclinical phase has been confirmed.

In a very recent research Wright et al. [38] also used the spatial-temporal partial least squares (PLS-ST), a multivariate analysis that improves the data derived from modern imaging techniques. Using data derived from all points earned, the ST-PLS allows a rigorous statistical evaluation of changes in the waveform and signal distribution related to retinal function. The results of the traditional techniques of analysis were compared with that of the ST-PLS: the first revealed, in subjects suffering from T1D without DR, variations of the IT but not of the amplitudes and, in addition, the spatial position of these changes has not been identified. In contrast, using the ST-PLS, researchers found significant variations between groups and they could highlight the spatial position of these changes on the retinal map, confirming that the changes in retinal function in DM occur before the onset of clinical disease.

The mfERG examination has proved to be very useful in the preclinical phase but less suitable in the follow-up of patients with DR after medical intervention or laser surgery, especially in comparison with other methods of examination such as the OCT [39] and the IVFA [40]. However many results are conflicting, also because of the subjects heterogeneity and the differences in the techniques used.

For example, Durukan et al. [41] found that mfERG cannot be performed to evaluate retinal functionality after the treatment of diabetic macular edema (DME) with intravitreal injections of triamcinolone acetonide, probably because of the irreversible macular damage.

On the other hand, Du et al. [42] have documented a reduction in the amplitude of P1 wave after a treatment with laser photocoagulation and Leozappa et al. [43] have evaluated the mfERG 1 week and 1, 3, and 6 months after surgery (standard three-port pars plana vitrectomy with peeling of inner limiting membrane) in 25 eyes of 21 patients with DME: both researches have considered mfERG useful for predicting functional prognosis.

## 5. The Pattern ERG (PERG)

The pattern electroretinogram (PERG) detects the functionality of the innermost retinal layers (ganglion cells and fibers) [28]. PERG is measured by using conjunctival or skin electrodes that do not alter vision, and visual stimulus is constituted by a structured pattern (typically a chessboard) in which white and black elements alternate with a regular frequency.

Recent researches showed PERG high sensitivity in detecting preclinical abnormalities related to diabetes. Caputo et al. [44] examined 42 patients with T1D with a number of microaneurysms (highlighted by fluorescein angiography) from 0 to 4 and a disease duration less than 11 years. None of the patients had concomitant ocular disease or systemic complications related to diabetes. PERG results showed the amplitude of N95 wave significantly reduced in diabetics compared to control subjects of the same age, and significant differences were found between controls and diabetics without retinopathy, controls, and diabetics with retinopathy and between diabetic patients with early retinopathy versus diabetics without retinal impairment. In addition, the amplitude resulting inversely correlated with the duration of the disease.

Because of the sensitivity of the method in detecting the activity of retinal ganglion cells, PERG has been also intensively used in diabetic subjects with suspected glaucoma or ocular hypertension [45]. The amplitude of N95 wave was altered in diabetic subjects with suspected glaucoma compared to controls, even when the visual field examination was normal.

A further application of this test, recently showed by Ozkiriş [46], was to evaluate the functional recovery after treatment of diabetic macular edema with intravitreal injections of bevacizumab. After 1 and 3 months, the author found an increase in both visual acuity and the amplitude of P50 wave in 35 eyes treated with bevacizumab at a concentration of 2.5 mg.

## 6. The Focal ERG (FERG)

The focal ERG, also called foveal ERG or focal macular ERG (fmacERG), is mainly used for the evaluation of foveal cones [47]. Usually it is registered in an on-off modulation at low (e.g., 8 Hz) and high frequency (e.g., 41.4 Hz).

Deschênes et al. [48] showed an increase of implicit time and a reduction in the amplitude of the FERG in 26 patients with T2D but without any ophthalmoscopic sign of retinopathy compared with 52 healthy controls. They also showed a significant correlation between these changes and the duration of the disease rather than the values of glycated hemoglobin (index of glycemic control).

Ghirlanda et al. [49], however, have undergone 60 subjects affected by T1D to the analysis of FERG using a small stimulus (9 degrees) and a frequency of 8 Hz. The analysis of harmonics revealed an alteration of F2 wave which resulted from reduced amplitude in diabetics with mild or even absent retinopathy compared to healthy controls of the same age. A statistically significant correlation with such alterations has been demonstrated both with the duration of the illness and with glycemic control.

## 7. Visual Evoked Potentials (VEP)

The visual evoked potentials (VEPs) are defined as changes in the bioelectric potentials of the occipital cortex evoked by visual stimuli. They are generated by complex neurosensory events related to the translation and transmission of nerve impulses along the optic tract, from the photoreceptors to the occipital cortex. They can be elicited with pattern or with flash stimuli.

As pointed out by the recommendations of the International Federation of Clinical Neurophysiology (IFCN) and International Society for Clinical Electrophysiology of Vision (ISCEV) [50], it is extremely important to use standardized methods in order to standardize and share data between individual laboratories (Table 2).

The pattern VEP is constituted by a set of electrical responses evoked by the variation of luminance contrast of a structural stimulus (typically a chessboard) projected on a TV screen and detected with specific electrodes placed on the scalp.

The flash VEP, instead, is constituted by a set of electrical responses evoked by a light stimulus of short duration and high intensity. The response of optic nerve fibers to this type of stimulus is different from response to a pattern stimulus: in this case it has nothing to do with the ability of discrimination (visual acuity) but more roughly leads information of brightness (magnocellular system) and movement.

DM affects both electrophysiological and psychophysical aspects of visual function. The main parameters of VEPs that can be evaluated are latency, amplitude, topography, and shape of the wave. Several external factors such as technical characteristics, cooperation of the patient, fixing, attention, sex, age, transparency of the optical mediums, and the size of the pupil may alter, more or less significantly, the examination. However, the amplitude and the latency of the P-100 wave are the most reliable indicators of clinically significant alterations of the visual pathway. A significant reduction in amplitude and increased latency of VEPs was found in both types of DM without signs of retinopathy. This denotes a functional neuronal loss before that anatomical abnormalities can be detected.

Several studies involving patients with various degrees of retinopathy found a strong correlation between retinal neovascularization (proliferative DR) and abnormal VEPs, attributed to a substantial damage of the ganglion cells and the retinal nerve fiber in diabetic subjects [51–53].

Heravian et al. [54] have recently emphasized the role of the VEPs in identifying signs of damage to the retinal ganglion cells before the onset of clinical signs of the disease in 40 diabetic patients including 20 subjects with nonproliferative diabetic retinopathy (NPDR) and 20 others without any retinopathy on fundus oculi and compared to 40 age- and sex-matched normal nondiabetic controls.

The pathophysiology of central nervous system dysfunction in patients with DM is not completely understood, but it certainly has a multifactorial etiology. Probably vascular and metabolic factors are involved similarly to what happens in diabetic peripheral neuropathy, in which the ischemia and reduced protein synthesis cause the loss of nerve fibers. In support of a common pathogenetic hypothesis between peripheral and central neuropathy, some authors argue that subjects with peripheral damage have abnormalities of the VEPs higher than those without signs of peripheral nerve involvement [52]. It also seems that such damage is related to duration of disease rather than glycemic control [55].

What appears quite clear is that the damage to central neurons is very early compared to the retinal one [56, 57].

Recently more complex methods such as multifocal VEP (mfVEP) have also been used to try to correlate the alteration of evoked potentials with specific retinal areas. Wolff et al. [58] found significant mfVEP implicit time (IT) differences between controls and all patients with diabetes, controls, and diabetics without retinopathy and between controls and diabetics with retinopathy. In the retinopathy group, ITs from zones with retinopathy were significantly longer than ITs from zones without retinopathy. The mfERG IT was more frequently abnormal than mfVEP IT. Considering those findings, it would be recommended to assist VEPs with flash and pattern electroretinogram (PERG) in order to confirm the existence of an involvement of the outer retina and therefore exclude a direct involvement of the inner retina and/or of the visual pathway.

## 8. Conclusions

Retinopathy, as a major complication of diabetes, has clearly an important role in the genesis of visual dysfunction. However, as has been widely documented, several anomalies occur in the retina and in visual pathways long before structural alterations may be clinically detected.

Visual abnormalities in diabetes must be approached in a broader sense, considering the visual function as a complex sensory system. The techniques described allow the evaluation of this system in the various stages of the visual process and have an important role in both in clinic and research settings. Complete knowledge of the function and the electrophysiology of neuroretina allows having a deeper understanding of the effects of diabetes on the central nervous system, area that in this field has traditionally received less “attention” than the peripheral ones.

The purpose of this small review is to enhance the use of these diagnostic methods in everyday clinical practice improving the approach to the patient care (Table 3).

For a long time a repeatable, cheap, quick, and objective test for the screening of DR has been searched. Although with some technical limitations and quite high costs, the ERG, and the study of oscillatory potentials and mfERG in particular, have definitely proved to be a valuable and objective tool for the early diagnosis of the disease and potentially for the ophthalmologic follow-up of the diabetic patient. VEP examination, with the analysis of the P-100 wave, assesses the visual function from the retina to the visual cortex and, therefore, provides important information about the function of the optic pathway.

The greatest and most regrettable limitation of these diagnostic techniques is represented by the still low uptake. It is hoped that in the nearest future such limitation will be overcome. The latest researches data presented in this review can encourage both the research and above all the use in daily clinical practice.

## Figures and Tables

**Table 1:** Standard full field ERG protocols and parameters according to ISCEV.

ERG test	Adaptation/time	Stimulus range (cd∗s∗m^−2^)	Interstimulus time (s)	Main physiological generator
Scotopic ERG	Dark adapted/≥20 min	0.02–0.03	2.0	b-wave: rods
Massive ERG	Dark adapted/≥20 min	6.7–8.4	10	a-wave: photoreceptors b-wave: bipolar cells
Oscillatory potentials	Dark adapted/≥20 min	6.7–8.4	10	Middle retinal layer and vascular function
Photopic ERG	Light adapted (30 cd∗m^−2^)/≥10 min	2.7–3.4	0.5	a-wave: cones b-wave: bipolar cells
Flicker-ERG	Light adapted (30 cd∗m^−2^)/≥10 min	2.7–3.4	0.030–0.036	Cones

**Table 2:** Standards for VEP assessment according to ISCEV.

	Field size (deg)	Stimulus type	Stimulation	Background luminance (cd∗m^−2^)	Contrast (%)	Presentation rate
Pattern stimulation	>15	Pattern reversal or onset/offset	Monocular	—	>75	<1–3 reversals or ≤2 onsets per second
Flash stimulation	>20	Standard luminance flash (2.7–3.4 cd∗s∗m^−2^)	Monocular (recommended)	15–30	—	<1.5 flashes per second

**Table 3:** Summary of the advantages (left side) and disadvantages (right) of electrophysiological techniques described in relation to DR. All techniques reported are noninvasive, safe, objective, and repeatable. Full knowledge and the use of them in clinical practice can provide useful information in preclinical evaluation, prognosis, and follow-up of DR.

ERG (OPs)	(i) Precociously altered in preclinical stage of DR. (ii) Can predict progression from nonproliferative to proliferative DR.	(i) Massive retinal response, not able to detect dysfunctions localized in a single small area.

Flicker-ERG	(i) Directly reduced in proportion to the degree of DR.	(i) Nonspecific.

MfERG	(i) Able to detect localized and minimal dysfunctions. (ii) Precociously altered in preclinical stage of DR. (iii) Can predict macular edema and functional prognosis.	(i) Not very suitable in advanced DR and/or in the follow-up after medical or laser interventions.

PERG	(i) Able to provide macular functionality assessment in preclinical and clinical stages of DR. (ii) Evaluates functional recovery after treatment (e.g., intravitreal).	(i) Responses being susceptible to artifacts. (ii) Influenced by visual acuity, fixing, optical correction, transparency of optical mediums, and patient cooperation.

FERG	(i) Precociously altered in DR.	(i) Nonspecific.

VEPs	(i) Provide a reliable and objective indicator of clinically significant alterations of the visual pathway. (ii) Able to evaluate central nervous system dysfunctions in patients with DM. (iii) Directly correlated to diabetic age. (iv) Directly correlated to severity of DR.	(i) Influenced by cooperation of the patient (fixing, attention), age, transparency of the optical mediums, and size of the pupil.
